# Effect of Different Combinations of Dietary Vitamin A, Protein Levels, and Monensin on Inflammatory Markers and Metabolites, Retinol-Binding Protein, and Retinoid Status in Periparturient Dairy Cows

**DOI:** 10.3390/ani11092605

**Published:** 2021-09-05

**Authors:** Bruna C. Agustinho, Kirk C. Ramsey, Chel Moore, Chia-Yu Tsai, Cynthia M. Scholte, Mark A. McGuire, Pedram Rezamand

**Affiliations:** 1 Department of Animal, Veterinary, and Food Sciences, University of Idaho, Moscow, ID 83844, USA; brunac@uidaho.edu (B.C.A.); kirkramseydvm@gmail.com (K.C.R.); tsai4799@vandals.uidaho.edu (C.-Y.T.); cy.scholte@gmail.com (C.M.S.); mmcguire@uidaho.edu (M.A.M.); 2 Elanco Animal Health, Greenfield, IN 46140, USA; chelmoore@nutriquest.com

**Keywords:** vitamin A, immune function, gene expression, mastitis, periparturient

## Abstract

**Simple Summary:**

The transition period is a challenging time, which combines a massive increase in nutrient requirements and leads to a negative energy balance. Therefore, disorders related to negative energy balance, such as ketosis, are more frequent. In addition, in this period, oxidative stress increases, favoring immune suppression functions and inflammation, which may lead to increased susceptibility to other diseases such as metritis and mastitis. Therefore, the combination of ionophores, such as monensin, that mitigate the accentuated negative energy balance; vitamin A, which plays an important role in supporting the immune system; and a high level of crude protein might improve immune parameters in dairy cows in the transition period. This study demonstrated that monensin and vitamin A supplementation and high crude protein levels enhanced some vitamin status and inflammatory markers when used during the late prepartum period.

**Abstract:**

The objective of this study was to determine the effect of feeding different combinations of dietary vitamin A supplementation (0 or 110 IU/kg body weight), protein (10.3% or 12.2%), and an ionophore (monensin at 0 or 400 mg/day) on retinoid metabolism and immune function of dairy cows. Eighty multiparous Holstein dairy cows were studied from d −35 to +21 relative to expected parturition in a complete randomized block design with a 2 × 2 × 2 factorial arrangement of treatments. The significance of treatments was declared at *p* ≤ 0.05. Dairy cows receiving high crude protein (CP) diets with monensin had a greater retinol-binding protein serum concentration than cows receiving high CP diets without monensin (*p* = 0.04). Animals supplemented with vitamin A showed lower SCC (*p* = 0.04) and a higher thiobarbituric acid reactive substances concentration (*p* = 0.06) than cows non-supplemented. Moreover, cows receiving low crude protein diets had a greater haptoglobin concentration (*p* = 0.01). In addition, cows fed a high crude protein diet had a greater TNF-α expression in peripheral blood mononuclear cells (*p* = 0.04). Animals fed diets without monensin had a greater serum haptoglobin on day 3 postpartum than those fed monensin (*p* = 0.01). Moreover, dietary vitamin A increased serum 13-*cis* retinoic acid postpartum. We conclude that vitamin A, crude protein levels, and monensin fed during the close-up period affect milk somatic cell count, some vitamin statuses, and inflammatory markers during early lactation.

## 1. Introduction

The transition period has been defined as approximately 3 weeks before to approximately 3 weeks after parturition [1]. Managing dairy cows during the transition period poses many challenges to the dairy industry. A primary challenge associated with the transition period is the massive increase in nutrient requirements through the end of gestation and initiating and maintaining lactation. It has been demonstrated that at 4 days postpartum, a healthy cow’s intake of net energy and metabolizable protein only accounts for 65% and 75%, respectively, of her total requirements [2]. In addition, during the transition period, intake is reduced because of the hormonal changes [3]. Therefore, an increase in nutrient requirements is typically not met by nutrient intake. The accentuated negative energy balance favors the occurrence of several metabolic disorders and diseases, such as ketosis and displacement of the abomasum [4,5].

Monensin is an ionophore that has been used in transition dairy cows to mitigate the consequences of the negative energy balance [6]. Monensin modulates the rumen microbiome, leading to more propionate production [7] and providing more energy to the animal because propionate increases hepatic gluconeogenesis [7]. In addition, monensin reduces the ketone body [8,9] in blood circulation, indicating a reduction in lipolysis. Moreover, monensin has been related to improvement in the immune system [10].

Furthermore, during the transition period, oxidative stress is increased, leading to immune suppression functions and inflammation, which may set the stage for other diseases such as metritis and mastitis [11]. Therefore, some vitamins, such as vitamin A, play an important role in supporting the immune system [12]. Vitamin A and its derivatives have been researched to understand their role in this critical period. Retinol, the most abundant form of vitamin A, is essential for growth, vision, immunity, and epithelial cell differentiation and proliferation [13,14]. However, it has been shown that during the transition period, plasma retinol concentrations decrease dramatically and do not regain until about 4 weeks postpartum [15]. In addition, the literature reported that retinol-binding protein (RBP) concentration in the serum of dairy cows might be modulated by the dietary crude protein concentration [16]. Blood vitamin A concentration has been associated with the udder health of dairy cows, more specifically, the severity of mastitis [17,18]. Additionally, retinol is related to the proliferation and apoptosis of cells in the mammary epithelial [19,20] and might affect the milk yield.

The meta-analysis by Husnain and Santos [21] showed that greater protein levels in prepartum diets could also increase pre- and postpartum intake and more milk, milk fat, and milk protein in primiparous cows. However, there is no evidence in the literature about a diet that combines the effects of a greater protein, vitamin A as a booster to the immune system, and monensin as an energy metabolism modulator. We hypothesized that vitamin A supplementation combined with a high protein level and monensin inclusion in the peripartum would provide a better nutrient input, increase retinol-binding protein leading to a greater retinol level, thus better health status of the mammary glands, as well as raising the delivery of retinol into the milk. Therefore, our objectives were to determine the effect of feeding a combination of dietary vitamin A, protein levels, and an ionophore on retinoid metabolism and the immune function of periparturient dairy cows.

## 2. Materials and Methods

### 2.1. Animals and Experimental Diets

All the animal procedures were approved by the Institutional Animal Care and Use Committee of the University of Idaho (IACUC protocol number 2014–78).

Ninety-four multiparous cows started the experiment from day −35 relative to the expected calving date to day 21 postpartum; however, 14 animals from different treatments were removed because of clinical hypocalcemia (*n* = 5), foot issues/lameness (*n* = 4), mastitis (*n* = 3), and extreme calving difficulty (*n* = 2). There was no relationship between the treatments and the incidence of health issues or calving difficulty because the affected dairy cows belonged randomly to different treatments (protein level: high, *n* = 7 vs. low, *n* = 7; vitamin A: no, *n* = 4 vs. yes, *n* = 10; monensin: no, *n* = 4 vs. yes, *n* = 10). Eighty multiparous Holstein cows that completed the experiment were included in the final analyses. Cows were assigned to a treatment group in a 2 × 2 × 2 factorial arrangement of treatments in a completely randomized block design. The factors consisted of dietary protein levels (10.3% or 12.2%), vitamin A levels (0 or 110 IU/kg body weight), and monensin levels (0 or 400 mg/day). The animals were fed twice daily, one of eight dry cow ration combinations from −35 d relative to the expected calving date (Table 1). The control treatment pre-parturition was designated as low protein, no monensin, no vitamin A. After parturition, all animals were fed with the same lactation diet, formulated according to NRC (2001) [22]. The postpartum diets consisted of 1.47 Mcal/ day, 16.8% crude protein, no monensin, and 72,000 IU/head per day of vitamin A in the form of a liquid supplement. 

Liver biopsy samples were taken between ribs 12 and 13 on days −35, −3, +3, +10, and +21 relative to parturition. Tissue samples were immediately frozen in liquid nitrogen and stored at −80 °C until processing. Blood samples were collected on days −35, −21, −14, −7, +1, +2, +3, +6, +9, +12, +15, +18, and +21 by coccygeal venipuncture using 10 mL blood vacutainer tubes (BD Diagnostics, Franklin Lakes, NJ, USA). Blood samples were centrifuged at 1500× *g* for 15 min at 4 °C, and the serum was stored at −80 °C until metabolites were analyzed. Additional blood samples were collected using vacutainer tubes and used to obtain Peripheral Blood Mononuclear Cells (PBMC). Composite milk samples were obtained at the first, second, and third milking postpartum and every 3 days thereafter. Milk samples were analyzed for somatic cell count (SCC; Washington DHIA, Burlington, WA, USA) and vitamin concentration.

### 2.2. Serum Vitamin Analyses

All vitamin analyses were conducted by reversed-phase HPLC (Waters e2695 Separation Module, Waters Corp., Milford, MA, USA) with a photodiode array detector (Waters 2998, Waters Corp., Milford, MA, USA), as described by Tsai et al. [23]. To determine serum 13-*cis* retinoic acid and all-*trans* retinoic acid concentrations, 400 μL serum samples were mixed with 420 μL of acetonitrile and 20 μL of acetic acid. One aliquot of 20 μL of retinol acetate (Sigma-Aldrich, St. Louis, MO, USA) was used as the internal standard. Samples were extracted twice with 1.5 mL of a mixture of 6.5:1.5 hexane: isopropanol and 0.2 mL of HPLC-grade water, and the hexane layer was removed via evaporation under a light flow of nitrogen gas. The residue was re-suspended in 200 μL of a 50:50 mixture of the mobile phase solutions, and 100 μL was injected into the instrument. Separation of retinoids was performed using an isocratic gradient on a Waters C18 CSH 3.5 μm column (4.6 mm × 75 mm, Waters Corp.). The mobile phase consisted of two solvents. Solvent A consisted of a 10 mM ammonium acetate solution in HPLC grade water, and Solvent B consisted of acetonitrile, isopropyl alcohol, and tetrahydrofuran (45:45:10) at a flow rate of 1 mL/min. 

Serum retinol, α-tocopherol, and β-carotene concentrations were determined similar to serum 13-*cis* retinoic acid and all-*trans* retinoic acid except retinol palmitate used as the internal standard, and samples were analyzed using a non-isocratic method. A single mobile phase consisting of acetonitrile, dichloromethane, methanol, and n-butanol (72:12:8:0.08) and a 1.25 mL/min flow rate was used.

Milk retinol, α-tocopherol, and β-carotene were determined in a similar procedure used to serum, except for the extraction. Milk samples were processed by mixing 2.5 mL of milk with 5 mL of 1% pyrogallic acid solution dissolved in 200 proof HPLC grade ethanol, then mixed with 10 mL of a 50% potassium hydroxide solution. Samples were placed in a water bath for 7 min at 70 °C, cooled down, posteriorly, 10 mL of HPLC grade Petroleum Ether was added, and samples were mechanically shaken at high speed for 10 min. Specifically, 15 mL of HPLC grade water were added to each sample, and the samples were centrifuged for 10 min. Once complete, 5 mL of the upper layer was extracted and evaporated under a light nitrogen gas flow. Samples were dissolved in 100 µL of HPLC grade methanol, 400 µL of starting mobile phase (as described in serum analysis), and 100 µL was injected into the instrument. 

### 2.3. Serum Metabolites

Serum haptoglobin concentration was determined using an enzymatic assay (Tridelta, Maynooth, Co., Kildare, Ireland). A total of 50 µL of the first reagent (stabilized hemoglobin) was added to 3.25 µL of serum, followed by 70 µL of the second reagent (chromogen). Samples were incubated for 5 min at room temperature, and the absorbance was determined in a spectrophotometer (Synergy 2 microplate spectrophotometer, BioTek, Winooski, VT, USA) at 600 nm. Concentrations were determined using a standard linear curve according to instructions provided by the manufacture. Serum RBP concentration was determined using a commercially available ELISA kit (BioSource, San Diego, CA, USA) according to instructions provided by the manufacturer. Briefly, 100 µL of sample was added to a pre-coated 96-well plate, and 50 µL of the conjugate (RBP4-HRP) was added to each well. The plate was incubated for 1 h at 37 °C. Upon completion of the incubation, each well was washed 5 times using a 1× wash solution. Posteriorly, 50 µL of solution A (substrate for HRP enzyme) and B (stop reagent) were added to each well, and the plate was covered and incubated for 15 min at 37 °C. After incubation, 50 µL of stop solution was added, and the absorbance was determined in a spectrophotometer (Synergy 2 microplate spectrophotometer, BioTek, Winooski, VT, USA) at 450 nm. The standard curve fit an exponential model using SAS, and concentration was determined for each sample. Serum concentration of malondialdehyde (MDA) was determined via colorimetric assay testing for thiobarbituric acid reactive species (TBARS; Caymen Chemical Company, Ann Arbor, MI, USA) according to the manufacturer’s instruction. A total of 50 µL of serum was mixed with 50 µL of sodium dodecyl sulfate (SDS) solution, and 2 mL of color reagent (thiobarbituric acid, acetic acid, and sodium hydroxide) were added to each sample. Samples were boiled for 1 h and immediately cooled on ice for 10 min. Samples were centrifuged, transferred on a 96 well plate, and absorbance determined in a spectrophotometer (Synergy 2 microplate spectrophotometer, BioTek, Winooski, VT, USA) at 532 nm. A standard linear curve was created, and MDA concentration was determined according to the standard curve. The intra- and inter-assays’ coefficients of variation were determined to serum metabolites and RBP, and when those coefficients were higher than 10%, the samples were re-analyzed.

### 2.4. Peripheral Blood Mononuclear Cells (PBMC) Isolation

Peripheral blood mononuclear cells (PBMC) were isolated, according to Scholte et al., [24] with modifications, from 50 mL of blood by gradient centrifugation using a mixture of Histopaque 1077 and 1119 (Sigma Aldrich, St. Louis, MO, USA). After centrifugation at 456× *g* for 1 h at room temperature, plasma was discarded, and the buffy coat and red blood cells were collected. Red blood cells were lysed using a 10% NaCl solution and water, then further washed with phosphate-buffered saline (PBS). Samples were separated, and PBMC was stored at −80 °C for further processing for gene expression.

### 2.5. Gene Expression Analysis

Total RNA was extracted from PBMC and liver using NucleoSpin^®^ RNA (Macherey Nagel, Düren, Germany), as described by the manufacturer. The RNA concentration was determined using NanoDrop ND-1000 (NanoDrop Technologies, Rockland, DE, USA) spectrophotometer. The complementary DNA (cDNA) synthesis was carried out using Applied Biosystems High-Capacity Reverse Transcription Kit (Applied Biosystems, Foster City, CA, USA), as described by the manufacturer. 

Real-time reverse-transcribed PCR was carried out in a 7500 Fast real-time PCR system (Applied Biosystems, Foster City, CA, USA) using custom-designed TaqMan MGB probes on targeted genes Interleukin-1beta (IL-1β), Interleukin-6 (IL-6), Tumor Necrosis Factor-alpha (TNF-α), and Intercellular Adhesion Molecule (ICAM) in PBMC. The bovine RPS9 and GAPDH were used as endogenous controls, also called housekeeping genes (Table 2). Reaction mixture included 2 μL of cDNA, 10 µL of TaqMan^®^ Universal Master Mix II with Uracil-N-Glycosylase (Applied Biosystems, Foster City, CA, USA), 1 μL of Applied Biosystems 20X custom primer probe mixture (Applied Biosystems, Foster City, CA, USA), and 7 μL of RNase-free water.

### 2.6. Statistical Analyses

The experimental design was a 2 × 2 × 2 factorial arrangement of treatments in a completely randomized block design. Pre-planned orthogonal contrasts were used to compare individual dietary main effects (protein, vitamin A, and monensin), as well as two-way interaction between the main factors on the response variables measured. Secondarily, variables were compared over time. Response variables were analyzed with repeated-measures ANOVA using the MIXED model procedure (Version 9.3, SAS Institute Inc., Cary, NC, USA). Sources of variation in the model included effects of main factors and 2-way interactions amongst the main effects. When analyzed over time, time was also included in the model. Significance was declared at *p* ≤ 0.05 with trends toward significance when *p* < 0.1. Separation of means was accomplished using the diff option within SAS to perform a pair-wise test between means. Intercept was designated as the random effect, with the subject being the cow. Several covariate structures, including compound symmetry, unstructured, autoregressive(1), variance components, and Toeplitz matrix, were tested; however, compound symmetry fit the model best, likely because sampling periods were not equidistant. Samples taken on periods previously listed in the sampling section were used for the analysis of treatment and time. Data are presented as least square means (LSM) ± standard errors of the mean (SEM). 

Gene expression data were analyzed using delta Ct values (Ct values normalized to the average of the endogenous control genes, GAPDH and RPS9) and are graphically presented as fold change (2-ΔΔCt) relative to the control treatment (low protein, no supplemental vitamin A, no monensin).

## 3. Results and Discussion

### 3.1. Milk Somatic Cell

**Figure 1:**
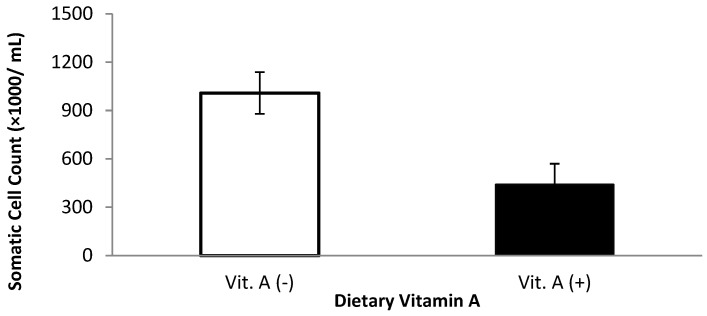
Somatic cell count for multiparous Holstein dairy cows (*n* = 80, total) that were fed diets with crude protein levels (10.3%, *n* = 40 vs. 12.2% dry matter basis, *n* = 40), and within each crude protein group that was fed monensin (400 mg/day per head or none) and vitamin A (110 IU/kg body weight or none) during prepartum period (from day −35 to the day of calving). All cows received a common lactation ration postpartum. Dietary vitamin A interaction (*p* = 0.04).

Milk somatic cell count and linear somatic cell score were affected by dietary treatments (Table 3). Milk somatic cell count was lower for cows that received vitamin A supplementation prepartum compared with those that did not receive vitamin A when the first, second, and third milkings were included in the analysis (*p* = 0.04, Figure 1). When the first three milkings were removed, there was a monensin × vitamin A interaction (*p* = 0.05); cows supplemented with vitamin A and monensin had the lowest SCC. The low SCC is possibly attributed to a combination of a balanced immune response (from vitamin A supplementation) combined with improved energy metabolism because of monensin.

Monensin and other ionophores have been related to reduced incidence of intramammary infection [25,26] in dairy cows, and this is likely related to an improvement in the immune function because of better energy metabolism [27]. Heuer et al. [25] noticed that cows in the first lactation were less predisposed to develop intramammary infections when treated with monensin before calving. This lower incidence in the literature is attributed to the better utilization of endogenous energy sources, leading to a better immune response and increased energy metabolites related to immunosuppression [28]. On the other hand, rats with inoculation of LPS in the mammary gland showed a greater migration of PMN to the tissue; however, when the treatment was combined with vitamin A supplementation, the effect of LPS was ameliorated [29]. Therefore, vitamin A has been related to the immune response in the mammary glands. This understanding may explain the monensin × vitamin A interaction and why cows not receiving vitamin A supplements had greater SCC overall. However, the results for SCC content contradict with observed by Oldham et al. [30] and Puvogel et al. [31], where SCC was not affected in dairy cows supplemented with vitamin A at 50,000 and 550,000 IU/day, respectively. Additionally, when β-carotene—a precursor of vitamin A—instead of vitamin A was supplemented in dairy cows at the prepartum, the SCC was not affected [32]. 

Similar results have also been observed using the rat model. *S. aureus* is the most common bacterium that causes mastitis [33], and Wiedermann et al. [34] observed that vitamin A deficiency predisposes rats to *S. aureus* infection. Wiedermann et al. [34] also indicated decreased phagocytic activity and bacterial killing of peritoneal macrophage for *S. aureus*. One of the main reasons neutrophil function might be diminished during vitamin A deficiency is that neutrophils and other granulocytes, developed from myeloid stem cells in the bone marrow, are mediated by retinoic-acid-binding to retinoic acid receptors to stimulate gene expression [35]. In general, SCC and SC linear score declined over the 21 d postpartum (data not shown), possibly related to the higher SCC at the beginning for the lactation. 

### 3.2. Milk Vitamins

Milk samples were tested over five time points (day 1, day 3, day 9, day 15, and day 21) during early lactation, and there was a reduction in milk vitamin A (*p* < 0.01; data not shown) and β-carotene (*p* < 0.01; data not shown) concentrations over time in postpartum. These reductions corroborate Johnston et al. [36] for milk vitamin A and β-carotene concentrations early postpartum. As reported in the literature, colostrum presents a greater concentration of vitamin A and E than milk [37]; therefore, the reduction observed in those vitamins early postpartum is possibly attributed to the transition from colostrum to milk. Furthermore, there was a protein × monensin interaction (*p* = 0.01, Table 4) for retinol concentration, where cows receiving low protein and no monensin had the lowest milk retinol concentration compared with all other treatment groups. 

**Figure 2:**
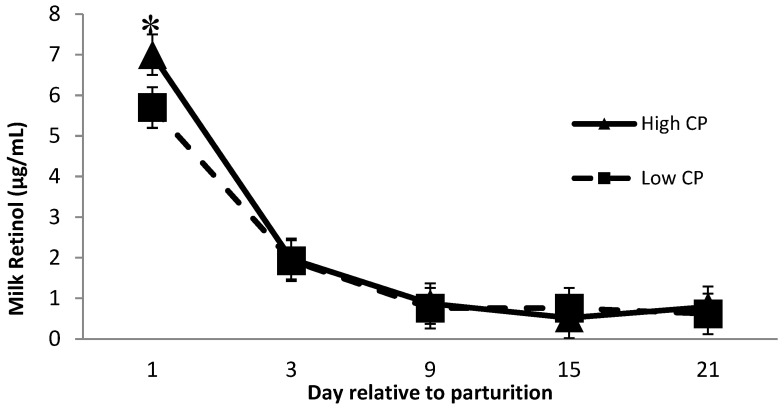
Milk retinol concentration the first 21 days of lactation for multiparous Holstein dairy cows (*n* = 80, total) that were fed diets with crude protein levels (10.3%, *n* = 40 vs. 12.2% dry matter basis, *n* = 40) and within each crude protein group that was fed monensin (400 mg/day per head or none) and vitamin A (110 IU/kg body weight or none) during prepartum period (from day −35 to the day of calving). All cows received a common lactation ration postpartum. Dietary crude protein × time interaction (*p* = 0.04). Asterisks show significant difference at time point specified.

Milk retinol was affected by dietary crude protein over time (Figure 2), where cows receiving high crude protein had greater (*p* = 0.04) milk retinol than cows receiving low crude protein 1 day after calving. Minimal research has been performed investigating the relationship between milk retinol and prepartum dietary crude protein. Lindberg et al. [16] reported a significant difference in serum RBP concentration between cows receiving low and high crude protein diets during the prepartum period and speculated that the effect of dietary protein levels on RBP might be related to the amino acid availability to synthesize RBP. This might explain the greater milk retinol concentration at day 1 postpartum from cows receiving high CP diets. Although, more investigations are required to better understand the potential effect of protein × monensin interaction on the milk retinol concentration. 

Milk α-tocopherol concentration was not affected by dietary treatments when observed over time (*p* = 0.80, data no shown). However, an interaction (*p* = 0.04) between dietary protein and monensin on milk α-tocopherol concentration was observed (Table 4). The animals fed diets with high crude protein combined with monensin showed a lower α-tocopherol concentration in milk than animals supplemented with high CP but no monensin (0.392 vs. 0.465 ug/mL). There is no evidence in the literature about the effect of high crude protein diets and monensin inclusion on milk α-tocopherol concentration; therefore, further investigation is required to better understand the potential impact. The β-carotene concentration in the milk was not affected among the treatments (*p* > 0.13). Milk α-tocopherol and β-carotene followed an expected curve over time, similar to what was observed with milk retinol concentrations. 

### 3.3. Serum Metabolites and Retinol-Binding Protein

**Figure 3:**
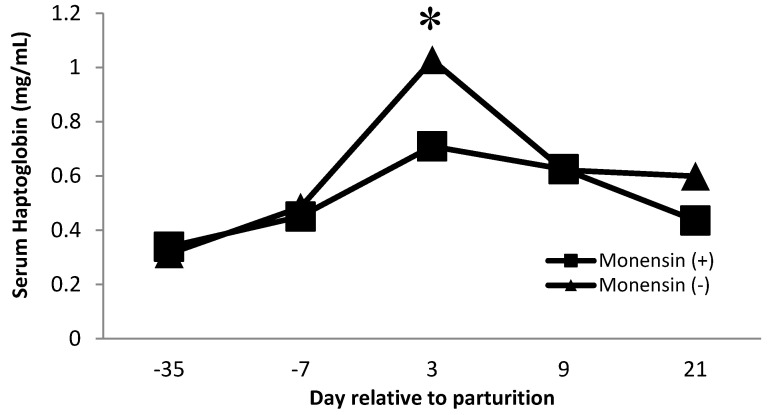
Serum haptoglobin concentration for multiparous Holstein dairy cows (*n* = 80, total) that were fed diets with crude protein levels (10.3%, *n* = 40 vs. 12.2% dry matter basis, *n* = 40), and within each crude protein group fed monensin (400 mg/day per head or none) and vitamin A (110 IU/kg body weight or none) during prepartum period (from day −35 to the day of calving). All cows received a common lactation ration postpartum. Dietary monensin × time interaction (*p* = 0.01). Asterisks show significant differences at time point specified.

Serum haptoglobin is an acute-phase protein that has been used as an inflammatory marker in dairy cows [38,39,40]. Haptoglobin acts by sequestering iron found in hemoglobin and avoiding the utilization of iron by bacteria. Furthermore, free hemoglobin promotes oxidation, so haptoglobin is also affected indirectly as an antioxidant [41]. Our observations follow similar observations in serum haptoglobin during the transition period, where serum haptoglobin concentrations increase near the time of parturition [40,42]. There was a monensin × time interaction (*p* = 0.01, Figure 3) on serum haptoglobin concentration. Cows that did not receive monensin had greater haptoglobin concentrations on day 3 postpartum than cows receiving monensin. The decrease in serum haptoglobin concentration for cattle receiving monensin may be partly related to the effect of monensin on changing rumen microflora. Monensin is known to increase the microbial population that produces propionate, improving energy metabolism [43,44]. The increase in glucose production may lead to fewer subclinical metabolic disorders such as hyperketonemia and fatty liver syndrome by decreasing negative energy balance during the transition period [44].

Furthermore, haptoglobin concentration is related to protein levels. Cows receiving low crude protein had greater (*p* = 0.01, Table 5) haptoglobin concentration than cows receiving high crude protein. There is no previous evidence in the literature about protein levels in the diet modifying haptoglobin concentration; therefore, further evidence is needed. It is important to recognize that haptoglobin is an acute-phase protein that is elevated during inflammation. Haptoglobin was measured to evaluate the effect of specific dietary factors that might improve the immune status of the animal. Therefore, a decrease in haptoglobin would be an indication of a lower immune system stimulation. In that regard, monensin and higher CP diets might lead to a better plane of nutrition that improves overall immune status. 

Lipid oxidation measured by TBARS assay determines the concentration of malondialdehyde (MDA) in the serum. Vitamin A supplementation affected serum MDA concentration (*p* = 0.06). The result indicates that cows receiving vitamin A tended to have a greater serum MDA than cows that did not. Although vitamin A and the β-carotene (precursor of vitamin A) have been shown to have antioxidant capacities [45], in the current study, vitamin A supplementation increased TBARS, an indicator of lipid oxidation. For instance, Dal-Pizzol et al. [46] reported an increase in lipid oxidation assessed through tests such as TBARS, superoxide dismutase, and others in rat Sertoli cells and concluded that retinol supplementation might cause this oxidation. Another study in the literature has shown similar results with retinol supplementation, concluding that retinol led to an increased TBARS and intracellular reactive species formation [47]. The current study provides evidence that vitamin A supplementation may have a deleterious effect on cellular oxidation; however, further investigation is required because serum MDA was the only measure used to determine the antioxidant capacity of cows in our experiment.

**Figure 4:**
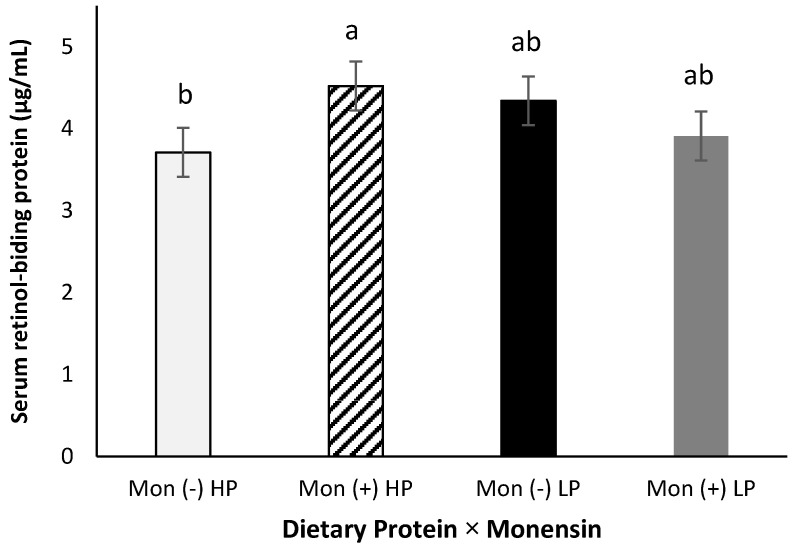
Serum retinol-binding protein (RBP) concentration for multiparous Holstein dairy cows (*n* = 80, total) that were fed diets with crude protein levels (10.3%, *n* = 40 vs. 12.2% dry matter basis, *n* = 40), and within each crude protein group fed monensin (400 mg/day per head or none) and vitamin A (110 IU/kg body weight or none) during prepartum period (from day −35 to the day of calving). All cows received a common lactation ration postpartum. Dietary protein × monensin (*p* < 0.04). Different letters mean statistical difference between the treatments.

Serum retinol-binding protein showed a protein × monensin interaction (*p* = 0.04, Figure 4), where cows fed with the combination of high CP and monensin showed a greater RBP concentration than cows fed with high CP and no monensin (4.52 vs. 3.71 µg/mL). This interaction might be related to combining a better amino acid availability (from the high CP) and energy metabolism (from monensin inclusion). Lindberg et al. [16] showed that a greater dietary protein increased hepatic transport proteins, such as RBP, indicating a positive impact on the synthesis and release. In the current study, serum RBP concentration was not significantly affected by dietary crude protein alone (*p* > 0.98). This difference between the results observed in the present study and the Lindberg et al. study might be attributed to the higher crude protein concentration in prepartum diets used by Lindberg et al. We [48] also did not observe a difference in serum RBP of rats fed different levels of dietary crude proteins supplemented with or without vitamin A.

Serum RBP concentrations were much lower than reported in the literature [49]. Abd Eldaim (2010) [49] reported an average concentration in early postpartum lactating dairy cows of 45 µg/mL using a Western blot analysis. The significantly lower RBP concentrations observed in the current study might be explained by the different methods used to determine RBP concentrations. Furthermore, Lindberg (1999) [16] also reported significantly greater serum RBP concentrations in periparturient multiparous and primiparous cows using a monoclonal antibody ELISA developed in their lab. The polyclonal antibody ELISA used in our determination of serum RBP may not be as sensitive as needed. Limited commercially available RBP ELISA are currently being produced, and further product development with greater sensitivity and specificity is required. Despite the lower serum RBP concentrations, a similar pattern in serum RBP concentrations is observed (data not shown) compared to the literature [16,49], with a reduced concentration close to the parturition. This reduction might be related to the RBP moving from serum into colostrum following other proteins passed from dam to offspring [49]. 

### 3.4. Serum Vitamins and Retinoid Concentrations

No significant difference was detected among diets on serum retinol concentrations (*p* > 0.27, Table 6). Lindberg et al. [16] observed that primiparous dairy cows supplemented with high crude protein in prepartum had a greater serum retinol concentration. In the current study, however, only multiparous cows were included. Furthermore, there was no significant difference in the interactions tested for serum retinol concentrations (*p* > 0.12). Our data follow the trend reported in the literature [17,30,50] in that serum retinol concentration decreased during the prepartum period (data not shown). Hydrolyzed retinyl-esters (RE) and carotenoids are taken up by the liver and stored in parenchymal cells and liver adipose cells. Stored RE is mobilized as retinol by a specific circulating protein called retinol-binding protein (RBP). Retinol-binding protein is responsible for the transportation of retinol to extrahepatic tissue throughout the body [51]. Retinol is initially absorbed by the lymphatic system, and excess is stored in the liver, and this may explain why an increase in retinol was not observed in serum during or immediately after supplementation. On the other hand, Pulvogel et al. [31] observed that cows supplemented with vitamin A (500,000 IU/day) during the dry period showed a greater concentration of retinol in the plasma at the end of the dry period and between d1 and d5 after calving compared with the animals in the control group. 

Similar to serum retinol concentration, no significant treatment effect was detected over time for either α-tocopherol or β-carotene (*p* = 0.96 and 0.98, respectively; data not shown). As reported by previous studies [17,50,52], both serum α-tocopherol and β-carotene decreased around the time close to the parturition and recovered postpartum. The dramatic decrease in serum vitamin concentration occurs likely because of the large requirements needed for colostrogenesis. This occurs via diet supplies, and stored lipid-soluble vitamins, such as vitamin A, are shunted toward the mammary gland [49]. 

**Figure 5:**
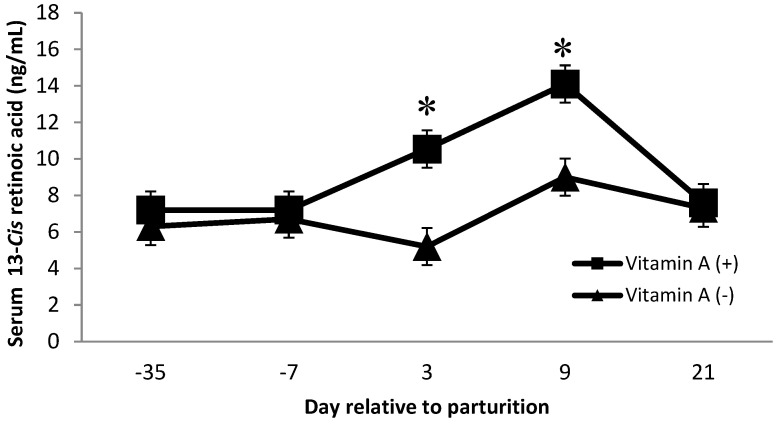
Serum 13-*Cis* retinoic acid concentrations (ng/mL) for multiparous Holstein dairy cows (*n* = 80, total) that were fed diets with crude protein levels (10.3%, *n* = 40 vs. 12.2% dry matter basis, *n* = 40), and within each crude protein group fed monensin (400 mg/day per head or none) and vitamin A (110 IU/kg body weight or none) during prepartum period (from day −35 to the day of calving). All cows received a common lactation ration postpartum. Dietary vitamin A × time interaction (*p* < 0.01). Asterisks show significant differences at time point specified.

Dietary factors contributed to changes in serum concentrations of 13-*cis* and all-*trans* retinoic acid. There was a vitamin A × protein interaction on 13-*cis* retinoic acid (*p* = 0.02), where cows receiving vitamin A and low crude protein showed a greater concentration. Furthermore, on day 3 and day 9 postpartum, cows receiving vitamin A supplementation had greater (*p* < 0.01) concentrations of 13-*cis* retinoic acid than cows that did not receive a vitamin A supplement (Figure 5). Van Merris et al. [14] demonstrated that nulliparous cows with experimentally induced *Eschericia coli* mastitis had significantly reduced serum concentrations of 13-*cis* retinoic acid. Therefore, we speculate that the increase in 13-*cis* retinoic acid at day 3 and day 9 for cows supplemented with vitamin A may have been related to the decrease in SCC for cows supplemented with vitamin A. However, further investigations are needed to better understand the interaction of dietary protein and vitamin A on 13-*cis* retinoic acid. In addition, diets did not alter serum 13-*cis* or all-*trans* retinoic acid concentrations over time (*p* = 0.30 and 0.80, respectively, data not shown).

**Figure 6:**
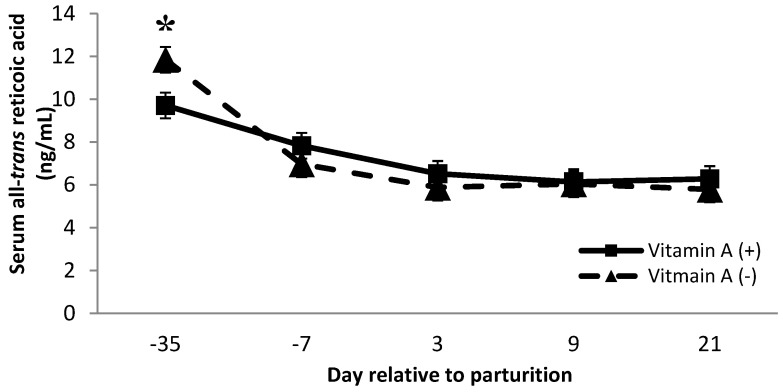
Serum all-*trans* retinoic acid concentration (ng/mL) for multiparous Holstein dairy cows (*n* = 80, total) that were fed diets with crude protein levels (10.3%, *n* = 40 vs. 12.2% dry matter basis, *n* = 40), and within each crude protein group fed monensin (400 mg/day per head or none) and vitamin A (110 IU/kg body weight or none) during prepartum period (from day −35 to the day of calving). All cows received a common lactation ration postpartum. Dietary vitamin A × time interaction (*p* = 0.005) (*p* < 0.01). Asterisks show significant differences at time point specified.

Cows receiving no vitamin A supplement had greater all-*trans* retinoic acid concentration than cows receiving the vitamin A supplement on d −35 (*p* < 0.01, Figure 6). Although all-*trans* retinoic acid might be related to increased SCC, the data from the present study do not confirm this connection because cows that did not receive vitamin A supplementation had significantly greater milk SCC (Table 1); there was no difference in all-*trans* retinoic over the time points analyzed, except for d −35. Somatic cell count is used to monitor mammary gland inflammation, and interestingly, the increase in SCC seen in the current study was observed in cows not receiving vitamin A supplementation. These results provide further evidence that not only does vitamin A play a role in immune function, but supplementation of vitamin A also improves mammary immune status. There were tendencies for protein × monensin interaction on 13-*cis* retinol acid and β-carotene serum concentration (*p* = 0.06; 0.09, respectively), where cows fed with low protein diets associated with monensin inclusion had a greater 13-*cis* retinol acid than animals fed with low protein without monensin or high protein with monensin. Moreover, cows fed with low protein diets with monensin had a greater β-carotene than cows fed with low protein without monensin. There is no evidence of similar effects in the literature; therefore, further investigations can help to better understand the interaction of these metabolites as both have important functions in immunity.

### 3.5. Gene Expression Analyses 

**Figure 7:**
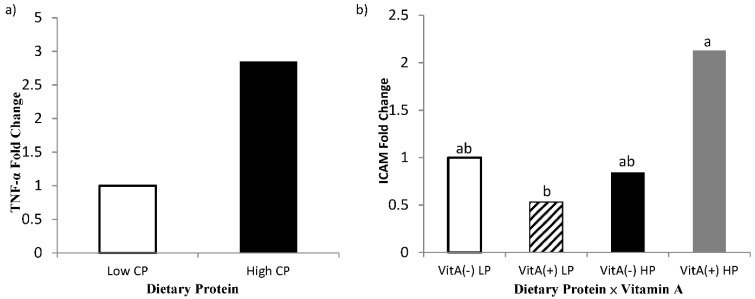
Fold change of PBMC gene expression of tumor necrosis factor-α (TNF-α; **Panel a**) and intercellular adhesion molecule-1 (ICAM; **Panel b**) for multiparous Holstein dairy cows (*n* = 80, total) that were fed diets with crude protein levels (10.3%, *n* = 40 *vs.* 12.2% dry matter basis, *n* = 40), and within each crude protein group fed monensin (400 mg/day per head or none) and vitamin A (110 IU/kg body weight or none) during prepartum period (from day −35 to the day of calving). All cows received a common lactation ration postpartum. Dietary crude protein effect and dietary protein × vitamin A interaction (*p* = 0.04, 0.05, respectively).

An effect of high crude protein on PBMC expression of TNF-α was observed in the present study, where cows receiving high crude protein had greater (*p* = 0.04, Figure 7) TNF-α gene expression. The interaction of protein by vitamin A on gene expression of ICAM in PBMC is shown in Figure 7, where cows receiving high crude protein and vitamin A supplement had a greater (*p* = 0.05, Table 7) expression of ICAM than the cows receiving vitamin A and low crude protein, requiring further investigations. Intracellular adhesion molecule (ICAM) expression is mediated by the expression and binding of both TNF-α and 9-*cis* retinoic acid, as demonstrated by Chadwick et al. [53] in immortalized human aortic endothelial cells. As both TNF-α and 9-*cis* retinoic acid were affected synergistically to aid in the expression of ICAM, it may explain why cows receiving vitamin A had greater expression of ICAM in isolated PBMCs.

The increase in TNF-α for cows receiving high crude protein may also provide evidence toward the increase in ICAM expression as ICAM is induced by TNF-α. According to the literature, ICAM is upregulated when retinoic acid is used as a treatment for cervical cancer in humans [54]. The upregulation suggests that retinol may enhance cellular emigration to sights of inflammation by upregulating the expression of ICAM in neutrophils of periparturient dairy cows. Therefore, this understanding provides evidence about why cows receiving vitamin A supplementation had lower SCC than cows not supplemented. It is important to note that greater ICAM expression resulted from the interactive effect of vitamin A supplementation and high crude protein diet. It is speculated that the greater crude protein diet may have aided immune status by improving the plane of nutrition for cows near the parturition combined with the role of vitamin A in the immune system. The better plane of nutrition may also explain the increase in TNF-α expression for cows receiving the higher crude protein diet. Gene expression of interleukin 1-β and IL-6 were also measured; however, no significance was detected among dietary treatments (*p* > 0.45) or interactions (*p* > 0.32). The hepatic gene expression of TNF-α and RBP were also determined; however, there was no difference between the treatment (*p* > 0.19; data not shown).

## 4. Conclusions

In summary, dairy cows supplemented with vitamin A during the late prepartum period showed a lower milk SCC postpartum and a greater serum TBARS concentration pre- and postpartum. Moreover,, high crude protein diets led to a greater milk retinol content postpartum and a greater gene expression of TNF-α in PBMC, and low crude protein diets resulted in a greater serum haptoglobin concentration pre- and postpartum. Furthermore, the combination of high protein and monensin increased serum retinol-binding protein. Further research is required to better understand how these factors mechanistically affect the immune system and how they can be used to decrease the incidences of both mammary gland infections and metabolic disorders.

## Figures and Tables

**Table 1:** Ingredient and chemical composition of the experimental diets.

Item	Ration		
	Prepartum (HP)	Prepartum (LP)	Postpartum
Ingredients (% of DM)			
Alfalfa hay	18.11	18.06	15.9
Grass hay	23.50	22.06	5.0
Wheat straw	11.01	10.15	-
Soybean, hulls	12.8	12.8	-
Soybean, meal	14.3	-	-
Rolled barley	4.68	9.42	17.0
Dry distiller corn grain with solubles	7.96	11.03	12.82
Corn, dry	3.12	11.84	15.32
Sodium bicarbonate	-	-	0.41
Liquid mineral/vitamin pre-mix ^1^	4.53	4.64	4.50
Calcium soaps of fatty acids ^2^	-		1.35
Triticale silage ^3^	-	-	13.01
Canola meal	-	-	14.99
Chemical analysis (% of DM)			
DM	59.10	57.28	63.83
CP	12.2	10.3	16.8
NDF	49.4	46.9	40.8
ADF	32.9	31.0	26.7
Ether extract	1.40	1.46	3.22
NFC	28.03	32.42	30.03
Ca	0.7	0.7	0.7
P	0.3	0.3	0.5
Mg	0.2	0.2	0.2
K	1.92	1.74	1.48
NE_L_, Mcal/kg of DM	1.34	1.38	1.47

^1^ Performix, Caldwell, ID. Vitamin A excluded in prepartum; ^2^ EnerGII; Virtus Nutrition, Corcoran, CA; ^3^ Triticale silage: 37% of DM (as fed). DM = dry matter; CP = crude protein; NDF = neutral fiber detergent; ADF = acid fiber detergent; NFC = non-fibrous carbohydrates.

**Table 2:** Custom bovine primer/probe sets used for reverse transcription-PCR.

Symbol	Amplicon Length (bp)	Accession No.	Primer (F, Forward; R, Reverse) and Probe	
IL-1β	69	NM_174093	F	GCTCTCCACCTCCTCTCACA
			R	CTCTCCTTGCACAAAGCTCATG
			Probe	CAGAACACCACTTCTCG
IL-6	65	NM_001015617	F	GGACGGATGCTTCCAATCTG
			R	GAAGACCAGCAGTGGTTCTGAT
			Probe	CAATCAGGCGATTTGC
TNF-α	82	NM_173966	F	GCTCTCTTGGCAGCTTTCCT
			R	GGCATCGAAGTTCTGTACTCATTCT
			Probe	CAGAACTGCAGCTTCAC
ICAM	73	NM_174348	F	GCAGGTGGTCCACAAACAC
			R	GCAATCCCGCTGGTCTAGTC
			Probe	ATGTCCTGTACGGCCCC
RPS9	71	XM_864261	F	GGCGGCTCGTCCGTATC
			R	AATCTTCAGGCCCAGGATGTAATC
			Probe	CCCTCATCCAGCACCC
GPDH	68	NM_001034034	F	GCTACACTGAGGACCAGGTT
			R	AGCATCGAAGGTAGAAGAGTGAGT
			Probe	CTCCTGCGACTTCAAC

IL-1β = Interleukin-1 beta; IL-6 = Interleukin-6; TNF-α = Tumor Necrosis Factor-alpha; ICAM = Intercellular Adhesion Molecule; RPS9 = Ribosomal protein subunit-9; GPDH = Gliceraldeído-3-fosfato desidrogenase.

**Table 3:** Milk somatic cell count and linear somatic cell score during the first 21 days of lactation for multiparous Holstein dairy cows (*n* = 80, total) that were fed diets with crude protein levels (10.3%, *n* = 40 vs. 12.2% dry matter basis, *n* = 40), and within each crude protein group fed monensin (400 mg/day per head or none) and vitamin A (110 IU/kg body weight or none) during prepartum period (from day −35 to the day of calving). All cows received a common lactation ration postpartum.

			Main Effect				SEM ^1^	*p*-Value					
	Vitamin A		Protein		Monensin								
Items	(+)	(−)	High	Low	(+)	(−)		Pro	Mon	Vit. A	Pro × Vit.	Mon × Vit.	Pro × Mon
SCC ^2^	706.9	1246	1003	949.5	1026	927.5	198.1	0.84	0.71	0.04	0.99	0.35	0.48
SCLs ^3^	2.77	3.09	2.94	2.91	2.99	2.86	0.15	0.86	0.52	0.11	0.99	0.34	0.50
* SCC ^2^	441.2	1008	786.4	663.6	637.8	812.1	129.2	0.47	0.31	<0.01	0.82	0.05	0.17
* SCLs ^3^	2.40	2.73	2.62	2.51	2.59	2.54	0.15	0.56	0.82	0.10	0.79	0.22	0.21

^1^ Largest SEM reported; ^2^ SCC: somatic cell count (×1000/mL); ^3^ SCLs: somatic cell linear score; * first, second, and third milk samples not included in analysis. Pro = dietary crude protein; Mon = monensin; Vit. A = dietary vitamin A; Pro × Vit. = dietary crude protein and vitamin A interaction; Mon × Vit. = monensin and dietary vitamin A interaction; Pro × Mon = dietary crude protein and monensin interaction.

**Table 4:** Milk vitamin concentrations (µg/mL) during the first 21 days of lactation for multiparous Holstein dairy cows (*n* = 80, total) that were fed diets with crude protein levels (10.3%, *n* = 40 vs. 12.2% dry matter basis, *n* = 40), and within each crude protein group that was fed monensin (400 mg/day per head or none) and vitamin A (110 IU/kg body weight or none) during prepartum period (from day −35 to the day of calving). All cows received a common lactation ration postpartum.

			Main Effect				SEM ^1^	*p*-Value					
	Vitamin A		Protein		Monensin								
	(+)	(−)	High	Low	(+)	(−)		Pro	Mon	Vit. A	Pro × Vit. A	Mon × Vit. A	Pro × Mon
Retinol	2.23	1.96	2.23	1.96	2.16	2.03	0.12	0.07	0.45	0.09	0.68	0.35	0.01
α-TOC	0.40	0.45	0.43	0.43	0.42	0.44	0.02	0.99	0.47	0.10	0.51	0.20	0.04
β-CAR	1.03	1.03	1.14	0.93	0.98	1.08	0.11	0.13	0.46	0.97	0.81	0.80	0.23

^1^ Largest SEM reported; α-TOC = α-tocopherol; β-CAR = β-carotene; Pro = dietary crude protein; Mon = monensin; Vit. A = dietary vitamin A; Pro × Vit. = dietary crude protein and vitamin A interaction; Mon × Vit. = monensin and dietary vitamin A interaction; Pro × Mon = dietary crude protein and monensin interaction.

**Table 5:** Serum metabolites for multiparous Holstein dairy cows (*n* = 80, total) that were fed diets with crude protein levels (10.3%, *n* = 40 vs. 12.2% dry matter basis, *n* = 40), and within each crude protein group fed monensin (400 mg/day per head or none) and vitamin A (110 IU/kg body weight or none) during prepartum period (from day −35 to the day of calving). All cows received a common lactation ration postpartum.

			Main Effect				SEM ^1^	*p*-Value					
	Vitamin A		Protein		Monensin								
	(+)	(−)	High	Low	(+)	(−)		Pro	Mon	Vit. A	Pro × Vit. A	Mon × Vit. A	Pro × Mon
Hpt ^2^	0.57	0.54	0.5	0.61	0.54	0.57	0.03	0.01	0.54	0.44	0.76	0.77	0.13
TBARS ^3^	2.22	2.01	2.15	2.08	2.07	2.16	0.08	0.53	0.43	0.06	0.77	0.24	0.14
RBP ^4^	4.16	4.08	4.11	4.13	4.21	4.02	0.21	0.97	0.53	0.80	0.92	0.70	0.04

^1^ Largest SEM reported; ^2^ expressed in mg/mL; ^3^ expressed in µM; ^4^ expressed in µg/mL; Hpt = haptoglobin; TBARS = thiobarbituric acid reactive species; RBP = retinol-binding protein; Pro = dietary crude protein; Mon = monensin; Vit. A = dietary vitamin A; Pro × Vit. = dietary crude protein and vitamin A interaction; Mon × Vit. = monensin and dietary vitamin A interaction; Pro × Mon = dietary crude protein and monensin interaction.

**Table 6:** Serum concentration for various retinoids, α-Tocopherol, and β-carotene of multiparous Holstein dairy cows (*n* = 80, total) that were fed diets with crude protein levels (10.3%, *n* = 40 vs. 12.2% dry matter basis, *n* = 40), and within each crude protein group fed monensin (400 mg/day per head or none) and vitamin A (110 IU/kg body weight or none) during prepartum period (from day −35 to the day of calving). All cows received a common lactation ration postpartum.

			Main Effect				SEM ^1^	*p*-Value					
	Vitamin A		Protein		Monensin								
Items	(+)	(−)	High	Low	(+)	(−)		Pro	Mon	Vit. A	Pro × Vit. A	Mon × Vit. A	Pro × Mon
Retinol (µg/mL)	2.10	1.92	2.03	2.00	1.96	2.06	0.12	0.82	0.52	0.27	0.12	0.69	0.94
*13-cis* RA (ng/mL)	9.42	7	7.9	8.5	8.6	7.8	0.41	0.24	0.19	<0.01	0.02	0.36	0.06
All-*trans* RA (ng/mL)	7.3	7.2	7.2	7.3	7.3	7.2	0.28	0.83	0.76	0.78	0.38	0.98	0.86
α-Tocopherol (µg/mL)	2.92	3.21	3.03	3.09	2.91	3.22	0.2	0.83	0.29	0.32	0.89	0.62	0.63
β-carotene (µg/mL)	12.9	12.3	13.3	11.9	12.6	12.7	1.66	0.75	0.82	0.60	0.56	0.78	0.09

^1^ Largest SEM reported; RA = retinoic acid; Pro = dietary crude protein; Mon = monensin; Vit. A = dietary vitamin A; Pro × Vit. = dietary crude protein and vitamin A interaction; Mon × Vit. = monensin and dietary vitamin A interaction; Pro × Mon = dietary crude protein and monensin interaction.

**Table 7:** PBMC gene expression (delta Ct) of interleukin 1 beta (IL1 β), interleukin 6 (IL6), tumor necrosis factor alpha (TNF-α), and intercellular adhesion molecule 1 (ICAM) for multiparous Holstein dairy cows (*n* = 80, total) that were fed diets with crude protein levels (10.3%, *n* = 40 vs. 12.2% dry matter basis, *n* = 40), and within each crude protein group fed monensin (400 mg/day per head or none) and vitamin A (110 IU/kg body weight or none) during prepartum period (from day −35 to the day of calving). All cows received a common lactation ration postpartum.

			Main Effect				SEM ^1^	*p*-Value					
	Vitamin A		Protein		Monensin								
Items ^2^	(+)	(−)	High	Low	(+)	(−)		Pro	Mon	Vit. A	Pro × Vit. A	Mon × Vit. A	Pro × Mon
IL1 β	4.84	5.28	4.81	5.31	4.91	5.21	0.71	0.54	0.71	0.59	0.32	0.74	0.65
IL6	13.2	12.5	13	12.7	12.8	12.9	0.86	0.77	0.84	0.45	0.73	0.67	0.32
TNF-α	4.62	5.06	4.09	5.6	4.34	5.34	0.62	0.04	0.17	0.54	0.07	0.15	0.23
ICAM	7.42	7.63	7.09	7.96	7.22	7.82	0.49	0.13	0.29	0.71	0.05	0.61	0.34

^1^ Largest SEM reported; ^2^ Gene expression data were analyzed using Ct values normalized to the average of the endogenous control genes, GAPDH and RPS9, and are presented graphically as fold change (2-ΔΔCt) relative to the control treatment. Pro = dietary crude protein; Mon = monensin; Vit. A = dietary vitamin A; Pro × Vit. = dietary crude protein and vitamin A interaction; Mon × Vit. = monensin and dietary vitamin A interaction; Pro × Mon = dietary crude protein and monensin interaction.

## Data Availability

The data presented in this study are available in Supplementary Material.

## Supplementary Materials

The following are available online at https://www.mdpi.com/article/10.3390/ani11092605/s1, Supplementary file: The data presented in this study.
